# The Associations of Periodontopathic Bacteria and Oral Candida with Periodontal Inflamed Surface Area in Older Adults Receiving Supportive Periodontal Therapy

**DOI:** 10.3390/diagnostics11081397

**Published:** 2021-08-02

**Authors:** Hideo Shigeishi, Mariko Nakamura, Iori Oka, Cheng-Yih Su, Kanako Yano, Momoko Ishikawa, Yoshino Kaneyasu, Masaru Sugiyama, Kouji Ohta

**Affiliations:** 1 Department of Public Oral Health, Program of Oral Health Sciences, Graduate School of Biomedical and Health Sciences, Hiroshima University, Hiroshima 734-8553, Japan; d203193@hiroshima-u.ac.jp (M.N.); m212911@hiroshima-u.ac.jp (I.O.); m190403@hiroshima-u.ac.jp (K.Y.); uknw0108moko@gmail.com (M.I.); yoshi-kane@hiroshima-u.ac.jp (Y.K.); masaru@hiroshima-u.ac.jp (M.S.); otkouji@hiroshima-u.ac.jp (K.O.); 2 Department of Oral Health Management, Program of Oral Health Sciences, Graduate School of Biomedical and Health Sciences, Hiroshima University, Hiroshima 734-8553, Japan; d181880@hiroshima-u.ac.jp

**Keywords:** periodontitis, Treponema denticola, Tannerella forsythia, Porphyromonas *gingivalis*, Candida albicans

## Abstract

The periodontal inflamed surface area (PISA) has been proposed for assessment of the total periodontal inflammatory status in people with periodontitis. This study was performed to investigate the associations of periodontopathic bacteria and candida with PISA in older people. We enrolled 100 patients aged ≥ 60 years who visited Hiroshima University Hospital. PISA and periodontal epithelial surface area (PESA) were calculated in each patient. Oral rinse samples were collected for DNA extraction. Periodontopathic bacteria and candida were detected by polymerase chain reaction. The mean values of PISA and PESA were significantly greater in *T.* *forsythia*-positive patients than in *T.* *forsythia*-negative patients. *T.* *forsythia/C. albicans* double-positive patients exhibited significantly greater PISA values than did non-double-positive patients. Additionally, PISA values were significantly greater in *T. forsythia//T. denticola/C. albicans* triple-positive patients than in *T. forsythia//T. denticola*/*C. albicans* non-triple-positive patients (*p* = 0.02). Propensity score-matching was performed between periodontopathic bacteria-positive and -negative patients using propensity scores generated from clinical factors. Importantly, *T.* *forsythia*/*T. denticola* double-positive patients exhibited significantly greater PISA values than non-double-positive patients among 72 propensity score-matched patients. Our preliminary results highlight the importance of the presence of *T.* *forsythia* and *T. denticola* for periodontal inflammation severity in older Japanese people.

## 1. Introduction

A dynamic balance between oral microorganisms and the host (involving genetics, host immune response, and circadian rhythm) is critical for the maintenance of oral health [1]. Periodontitis is a polymicrobial infectious disease mainly mediated by dysbiosis of the subgingival microbiota [2]. Specific complexes of bacteria are important for the onset of periodontitis [3]. So-called red complex bacteria such as *Treponema denticola*, *Tannerella* *forsythia*, and *Porphyromonas* *gingivalis* are associated with severe periodontitis [3]. Both *P. gingivalis* and *T. denticola* are present in subgingival plaque at deep periodontal pockets [4]. Microbial colonization with red complex bacteria is preceded by colonization with so-called orange complex bacteria (i.e., *Fusobacterium* subspecies, *Prevotella intermedia*, *Prevotella nigrescens, Peptostreptococcus micros*, *Campylobacter showae*, *Campylobacter gracilis*, *Campylobacter rectus*, *Streptococcus constellatus*, and *Eubacterium nodatum*) [3]. These red and orange complex bacteria have predominant roles in the etiology of periodontitis. *Aggregatibacter actinomycetemcomitans* is an important pathogenic species associated with localized aggressive periodontitis [5]. In addition, the presence of oral candida species is related to periodontitis [6,7]. Therefore, both periodontopathic bacteria and oral candida are presumed to initiate active inflammation in periodontal tissues.

The periodontal inflamed surface area (PISA) has been proposed for assessment of the total periodontal inflammatory status in people with periodontitis [8]. PISA is calculated by measuring clinical attachment level, recession, and bleeding on probing; this measurement reflects the surface area of bleeding pocket epithelium in square millimeters [8]. Bleeding on probing is regarded as a vital sign of the presence of local periodontal inflammation [8]. Therefore, PISA can be employed to quantitatively assess the inflammatory burden of periodontitis. However, the associations of periodontopathic bacteria and oral candida with PISA have not been fully elucidated in older Japanese people. The objective of this study was to investigate the associations of periodontopathic bacteria (e.g., *P. gingivalis*, *T. forsythia*, and *T. denticola*) and *Candida albicans* with PISA in older people.

## 2. Materials and Methods

### 2.1. Patients

**Figure 1:**
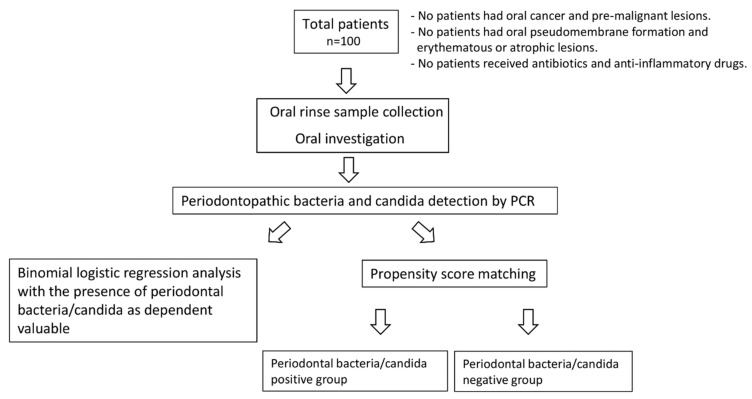
Schematic diagram of this study.

In this study, we enrolled 100 patients aged ≥ 60 years who visited Hiroshima University Hospital from 2018 to 2019 and received supportive periodontal maintenance therapy. No patients had oral lesions, such as oral cancer or pre-malignant lesion (i.e., leukoplakia). In addition, no patients had oral pseudomembrane formation and erythematous or atrophic lesions, which are characteristic findings of oral candidiasis. No patients received antibiotics or anti-inflammatory drugs (e.g., steroids) at the time of examination. The study protocol was approved by the Ethical Committee of Hiroshima University (approval no. E-1115) and all patients provided written informed consent to participate. A schematic diagram of this study is shown in Figure 1. Medical comorbidities, including cardiovascular diseases (e.g., heart disease and arrhythmia), hypertension, diabetes, and dyslipidemia, were among the clinical factors assessed in this study. Regarding smoking status, we divided patients into three groups: non-smokers (people who had never smoked in their lifetime), current smokers (people who had smoked daily or occasionally), and former smokers (people who had smoked in their lifetime, but who had quit smoking before the examination).

### 2.2. Oral Rinse Sample Collection and DNA Extraction

Patients were asked to rinse their mouths with saline for 15 s, then expel the rinse samples into sterile plastic tubes. Collected samples were centrifuged and supernatants were decanted; the resulting pellets were stored at −80 °C. DNA was extracted using a PureLink™ Microbiome DNA Purification kit (Thermo Fisher Scientific, Waltham, MA, USA), in accordance with the manufacturer’s protocol.

### 2.3. Oral Investigation

Probing depth and bleeding on probing were examined at six sites (i.e., mesiobuccal, mesiolingual, buccal, lingual, distobuccal, and distolingual) for each individual tooth using a periodontal probe (CP10 color coded probe, Hu-Friedy). Bleeding on probing was considered positive when gingival bleeding was observed within 20 s after probing [9]. Examination of probing depth and bleeding on probing was performed by an experienced dentist. Intra-rater reproducibility of pocket probing measurement by the dentist was investigated. The value of the intraclass correlation coefficient was 0.84. The patient’s periodontal condition is summarized in Table 1. The PISA and periodontal epithelial surface area (PESA) values were used as indicators of periodontal inflammatory status. PISA and PESA were calculated in accordance with a previously described method [8].

### 2.4. Periodontopathic Bacteria and Candida Detection by Polymerase Chain Reaction (PCR)

*P. gingivalis*, *T. forsythia*, *T. denticola*, and *C. albicans* were detected by PCR. The following previously reported PCR primer sets were employed in this study: *P. gingivalis*, 5′-AGGCAGCTTGCCATACTGCG-3′ (sense) and 5′-ACTGTTAGCAACTACCGATGT-3′ (antisense); *T. forsythia*, 5′-GCGTATGTAACCTGCCCGCA-3′ (sense) and 5′-TGCTTCAGTGTCAGTTATACCT-3′ (antisense); *T. denticola*, 5′-TAATACCGAATGTGCTCATTTACAT-3′ (sense) and 5′-TCAAAGAAGCATTCCCTCTTCTTCTTA-3′ (antisense); *C. albicans*, 5′-TTTATCAACTTGTCACACCAGA-3′ (sense) and 5′-ATCCCGCCTTACCACTACCG-3′ (antisense); and universal primers for the bacterial 16S ribosomal RNA gene, 5′-CGCTAGTAATCGTGGATCAGAATG-3′ (sense) and 5′-TGTGACGGGCGGTGTGTA-3′ (antisense) [10,11,12]. GoTaq^®^ Green Master Mix (Promega, Madison, WI, USA) was employed to amplify PCR products. Amplifications of PCR products were performed using an Eppendorf Mastercycler EP Gradient S Thermal Cycler (Eppendorf, Hamburg, Germany). The PCR protocol was as follows: 95 °C for 2 min, followed by 30 cycles of 95 °C for 1 min, 58 °C for 1 min, and 72 °C for 1 min. PCR products were electrophoresed on 2% agarose gels with ethidium bromide and were detected using an ultraviolet transilluminator. The presence of PCR products of expected sizes was determined by comparison with a 100 bp DNA ladder (Toyobo, Osaka, Japan).

### 2.5. Statistical Analysis

The Mann–Whitney U test or Kruskal–Wallis test was used to compare continuous numerical variables between groups. The χ^2^ test or Fisher’s exact test was used to compare categorical variables between groups. Binomial logistic regression analysis was conducted using a forced entry method. Binomial logistic regression analysis was performed using the presence of periodontal bacteria/candida as the dependent variable and variables with a *p*-value of <0.20 in univariate analysis as independent variables. A propensity score-matched analysis was performed to eliminate the effects of clinical confounding factors. Propensity scores were calculated by logistic regression analysis of eight clinical parameters (i.e., age and sex; cardiovascular disease, hypertension, diabetes, dyslipidemia, and smoking statuses; and number of remaining teeth). IBM SPSS Statistics, version 24.0 (IBM Corp., Armonk, NY, USA) was used for statistical analysis. To calculate the sample size of propensity score-matched patients required for paired *t*-tests, G*Power (version 3.1.9.4, Heinrich-Heine-Universität Düsseldorf, Düsseldorf, Germany) was used with an effect size of 0.5, 80% statistical power, and a significance level of 0.05. The minimum sample size required for paired t-tests was 34 patients per group. For all analyses, *p* < 0.05 was considered statistically significant.

## 3. Results

### 3.1. Associations of PISA and PESA with Clinical Factors in Older People

**Figure 2:**
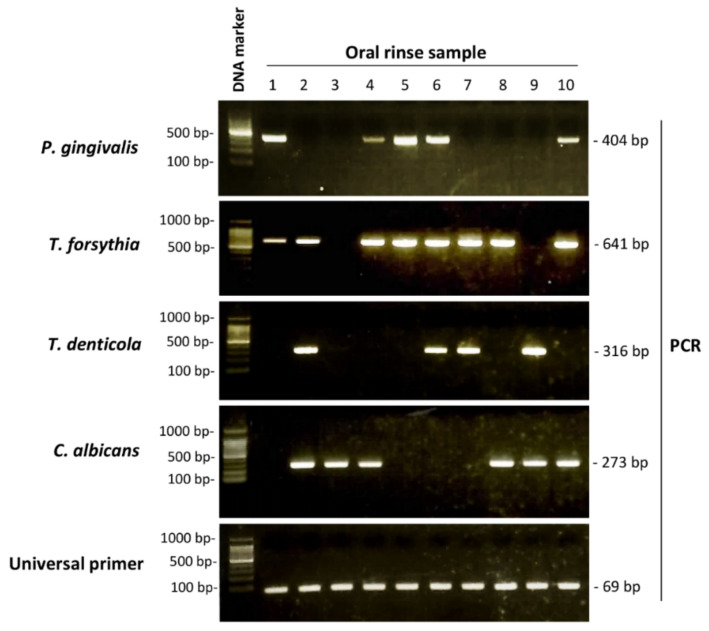
Polymerase chain reaction (PCR) detection of periodontopathic bacteria and candida. PCR was performed to detect DNA from *P. gingivalis*, *T. forsythia*, *T. denticola*, and *C. albicans*. PCR products were electrophoresed on 2% agarose gels, stained with ethidium bromide, and exposed to ultraviolet light using an ultraviolet transilluminator.

Amplified PCR products indicating the presence of *P. gingivalis*, *T. forsythia*, *T. denticola*, and *C. albicans* are shown in Figure 2. Of the 100 patients in this study, *P. gingivalis* was detected in 56 (56.0%)*, T. forsythia* was detected in 83 (83.0%), *T. denticola* was detected in 49 (49.0%), and *C. albicans* was detected in 39 (39.0%). Table 2 summarizes the associations of PISA and PESA with clinical factors. PESA values were significantly greater in smokers than in non-smokers (*p* = 0.04). Moreover, PISA values were greater in patients with diabetes or hypertension than in patients without those comorbidities. However, no significant associations were found between PISA and clinical factors.

### 3.2. Associations of the Presence of Periodontopathic Bacteria with PISA and PESA in Older People

**Figure 3:**
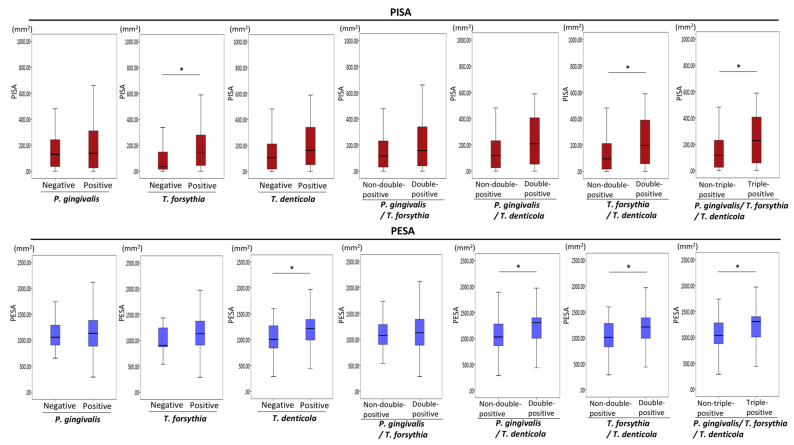
Associations of periodontopathic bacteria with PISA and PESA. Mann–Whitney U test. Statistical significance levels of *p* < 0.05 are indicated by *.

Figure 3 summarizes the associations of periodontopathic bacteria with PISA and PESA. The mean values of PISA and PESA tended to be greater in patients with *P. gingivalis* than in patients without *P. gingivalis*; however, these differences were not statistically significant. PISA values were significantly greater in patients with *T. forsythia* than in patients without *T. forsythia* (*p* = 0.01). The mean values of PISA tended to be greater in patients with *T. denticola* than in patients without *T. denticola*; however, this difference was not statistically significant. PISA values were significantly greater in *T. forsythia*/*T. denticola* double-positive patients than in *T. forsythia*/*T. denticola* non-double-positive patients (i.e., both double-negative and single-positive patients) (*p* = 0.01). In addition, PISA values were significantly greater in *P. gingivalis*/*T. forsythia*/*T. denticola* triple-positive patients than in non-*P. gingivalis*/*T. forsythia/T. denticola* triple-positive patients (i.e., triple-negative, single-positive, or double-positive patients) (*p* = 0.03). Significant differences in PESA were found between *T. denticola*-positive and -negative patients (*p* = 0.02), between *P. gingivalis*/*T. denticola* double-positive patients and non-*P. gingivalis*/*T. denticola* double-positive patients (*p* = 0.02), between *T. forsythia/T. denticola* double-positive patients and non-*T. forsythia/T. denticola* double-positive patients (*p* = 0.02), and between *P. gingivalis/T. forsythia/T. denticola* triple-positive patients and non-*P. gingivalis/T. forsythia/T. denticola* triple-positive patients (*p* = 0.03).

### 3.3. Associations of the Combined Presence of Periodontopathic Bacteria and C. albicans with PISA in Older People

**Figure 4:**
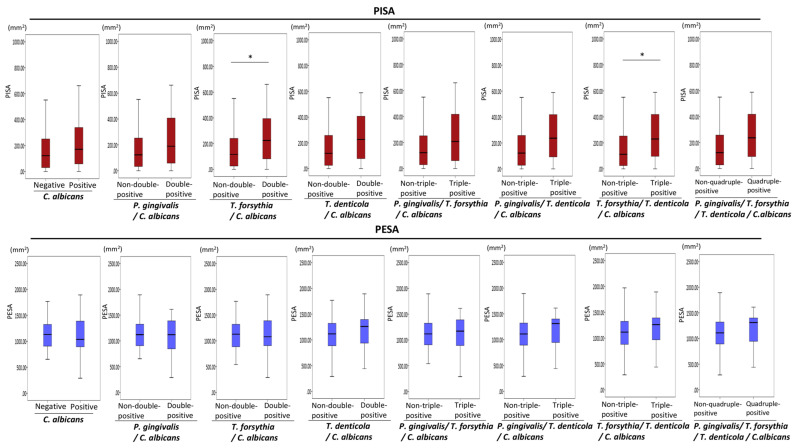
Associations of the combined presence of periodontopathic bacteria and candida with PISA and PESA. Mann–Whitney U test. Statistical significance levels of *p* < 0.05 are indicated by *.

No significant associations were found between *C. albicans* and PISA or PESA values (Figure 4). Next, the associations of the combined presence of periodontopathic bacteria and *C. albicans* with PISA and PESA were investigated. PISA values were greater in periodontopathic bacteria/*C. albicans* double-positive patients than in periodontopathic bacteria/*C. albicans* non-double-positive patients (Figure 4). PISA values were significantly greater in *T. forsythia*/*C. albicans* double-positive patients than in *T. forsythia*/*C. albicans* non-double-positive patients (*p* = 0.04) (Figure 4). Notably, PISA values were significantly greater in *T. forsythia*//*T. denticola*/*C. albicans* triple-positive patients than in *T. forsythia*//*T. denticola*/*C. albicans* non-triple-positive patients (*p* = 0.02) (Figure 4).

### 3.4. Associations of the Presence of Periodontopathic Bacteria/Candida with Clinical Factors

The results of binomial logistic regression analysis with the presence of periodontal bacteria/candida as dependent valuable are shown in Supplementary Tables S1–S15. Binomial logistic regression analysis revealed that the presence of *T. denticola* was significantly associated with PESA (odds ratio = 1.00, *p* = 0.048). The presence of *P. gingivalis*/*C. albicans* was significantly associated with the number of remaining teeth (odds ratio = 0.91, *p* = 0.03). Additionally, the presence of *P. gingivalis*/*T. forsythia*/*C. albicans* was significantly associated with the number of remaining teeth (odds ratio = 0.91, *p* = 0.03). However, no statistically significant relationship was observed between the presence of periodontal bacteria/candida and PISA.

### 3.5. Associations of the Presence of Periodontopathic Bacteria with PISA and PESA in Propensity Score-Matched Patients

Propensity score-matching was performed between periodontopathic bacteria-positive and -negative patients using propensity scores generated from eight clinical factors, as described in the Materials and Methods section. However, sample size requirements were not met for patients in the *T. forsythia*-positive versus *T. forsythia*-negative group (15 patients), *T. denticola* positive versus *T. denticola* negative group (33 patients), *P. gingivalis*/*T. denticola* double-positive group versus *P. gingivalis*/*T. denticola* non-double-positive group (29 patients), *P. gingivalis*/*T. forsythia*/*T. denticola* triple-positive group versus *P. gingivalis*/*T. forsythia/T. denticola* non-triple-positive group (29 patients), *P. gingivalis*/*C. albicans* double-positive group versus *P. gingivalis*/*C. albicans* non-double-positive group (17 patients), *T. forsythia*/*C. albicans* double-positive group versus *T. forsythia*/*C. albicans* non-double-positive group (29 patients), *T. denticola*/*C. albicans* double-positive group versus *T. denticola*/*C. albicans* non-double-positive group (21 patients), *P. gingivalis*/*T. forsythia*/*C. albicans* triple-positive group versus *P. gingivalis*/*T. forsythia*/*C. albicans* non-triple-positive group (20 patients), *P. gingivalis*/*T. denticola*/*C. albicans* triple-positive group versus *P. gingivalis*/*T. denticola*/*C. albicans* non-triple-positive group (16 patients), *T. forsythia*/*T. denticola*/*C. albicans* triple-positive group versus *T. forsythia*/*T. denticola*/*C. albicans* non-triple-positive group (21 patients), and *P. gingivalis*/*T. forsythia*/*T. denticola/C. albicans* quadruple-positive group versus *P. gingivalis*/*T. forsythia*/*T. denticola*/*C. albicans* non-quadruple-positive group (16 patients). Therefore, these groups were not included in further analyses.

**Figure 5:**
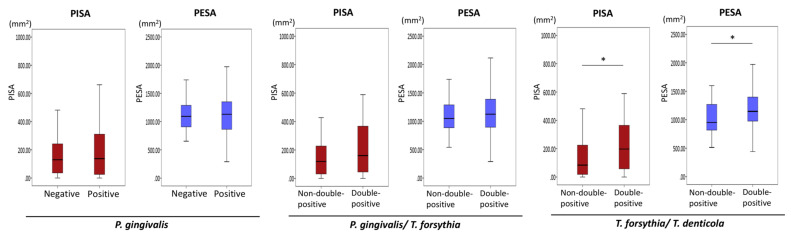
Associations of periodontopathic bacteria with PISA and PESA in propensity score-matched patients. Mann–Whitney U test. Statistical significance levels of *p* < 0.05 are indicated by *.

Finally, the associations of the presence of *P. gingivalis, P. gingivalis*/*T. forsythia,* and *T. forsythia*/*T. denticola* with PISA were investigated in propensity score-matched patients. None of the eight clinical variables were significantly associated with the presence of periodontopathic bacteria (Table 3, Table 4 and Table 5). Thirty-six *P. gingivalis*-positive patients exhibited greater PISA values than *P. gingivalis*-negative patients. However, the presence of *P. gingivalis* was not significantly associated with PISA (Table 3, Figure 5). In addition, *P. gingivalis* and *T. forsythia* double-positivity was not significantly associated with PISA in 72 propensity score-matched patients (Table 4, Figure 5). Importantly, 36 *T. forsythia* and *T. denticola* double-positive patients exhibited significantly greater PISA and PESA values than *T. forsythia* and *T. denticola* non-double-positive patients (*p* = 0.02 and *p* = 0.03, respectively) (Table 5, Figure 5).

## 4. Discussion

Significant correlations have been found between PISA and the severity of periodontitis or periodontal indexes (e.g., bleeding on probing and plaque accumulation) in systemically healthy patients with periodontitis [13]. To the best of our knowledge, no other studies have investigated the associations of periodontopathic bacteria and oral candida with PISA in Japanese older people. In this study, propensity score-matching analysis revealed that PISA values were greater in the *T. forsythia*/*T. denticola* double-positive group than in the *T. forsythia*/*T. denticola* non-double-positive group. Additionally, PISA values tended to be greater in the *P. gingivalis*/*T. forsythia* double-positive group than in the *P. gingivalis*/*T. forsythia* non-double-positive group, but this difference was not statistically significant. These results suggest that co-infection with *T. forsythia* and *T. denticola* is a critical factor associated with chronic periodontal inflammation in Japanese older people.

*T. denticola* is periodontal pathogen that contributes to the severity of periodontitis by enhancement of alveolar bone resorption [14]. *T. denticola* has several virulence factors such as dentilisin [15], the major surface protein [16], and factor H-binding protein B [17], which have been implicated in the progression of periodontitis. Importantly, the major surface protein of *T. denticola* plays a role in bacterial adherence to epithelial cells and components of the extracellular matrix [18]. Additionally, *T. forsythia* has a variety of notable virulence factors, including Bacteroides surface protein A [19], a trypsin-like protease [20], glucosidases [21], forsythia detaching factor [22], and hemagglutinin [23]. *T. forsythia* is frequently present along with *P. gingivalis* in patients who exhibit active periodontitis [24,25]. The growth of *T. forsythia* is accelerated when it is co-cultured with *F. nucleatum* [26]. Notably, the virulence of *T. forsythia* lipopolysaccharide was weakened upon co-culture with *P. gingivalis* [27]. These results imply that the virulence of *T. forsythia* is affected by co-infection with other periodontopathic bacteria. Lanza et al. reported that *T. denticola* was associated with greater gingival bleeding, while *T. forsythia* was associated with chronic periodontitis severity, suggesting that *T. denticola* and *T. forsythia* may be the main red complex members involved in periodontitis progression [28].

Virulence factors present within *Candida* species include extracellular hydrolytic enzymes (i.e., phospholipase, proteinases, lipases, and coagulase) [29]. Phospholipase enzyme digests membrane phospholipids of the host cell, thereby promoting cell lysis and subsequent infection [29]. *C. albicans*-produced metallopeptidase presumably aids in periodontitis progression by degrading the extracellular matrix [30]. In this study, PISA values were significantly greater in the *T. forsythia*/*C. albicans* double-positive group than in the *T. forsythia*/*C. albicans* non-double-positive group. Additionally, *T. forsythia*//*T. denticola*/*C. albicans* triple-positive patients showed significantly greater PISA values than non-triple-positive patients. Although the biological association between periodontopathic bacteria and *C. albicans* remains unknown, *C. albicans* may enhance periodontal inflammation in the presence of periodontopathic bacteria.

The presence of inflamed and ulcerated subgingival epithelium in the periodontal pocket provides the opportunity for bacterial endotoxins to disseminate into the bloodstream, which triggers inflammatory processes and the impairment of distant organs [31]. Thus far, multiple studies have shown associations between PISA and systemic diseases [32,33,34,35]. PISA was identified as a significant predictor of HbA1c level in healthy people without diabetes [32]. Diabetes is a known risk factor for periodontitis [36]. Although no significant association was observed between diabetes and PISA in this study, PISA values were greater in patients with diabetes than in patients without diabetes, indicating that diabetes is a major risk factor for periodontitis. Elevated PISA values and bleeding on probing have been associated with high blood pressure [33]. A 5-year longitudinal study revealed that greater PISA values were significantly associated with an elevated incidence of mild cognitive impairment in community-dwelling older people, suggesting that chronic periodontal inflammation contributes to the deterioration of cognitive function in older people [34]. Finally, elevated PISA values were independently predictive of poor prognosis in patients with lacunar infarcts [35]. These results suggest that elevated PISA values are associated with periodontitis severity and the presence of systemic disease; thus, PISA may be a valuable prognostic factor for some lifestyle-related diseases.

This study had an important limitation in terms of the sampling method; namely, oral rinse samples contain bacteria derived from various sites in the oral cavity. Thus, subgingival plaque and crevicular fluid samples may be more appropriate to determine the presence of periodontopathogens in the periodontal pocket. Further studies are needed regarding the relationship between periodontopathogens and periodontal inflammation to enable generalization of our findings.

## 5. Conclusions

*T. forsythia/T. denticola* co-infection was significantly associated with greater PISA values in older Japanese people after adjustment for potentially clinical confounding factors using a propensity score-matching method. *T. forsythia* and *T. denticola* are involved in chronic periodontal inflammation in older people. The preliminary results in this study highlight the important roles of several periodontal pathogens in severe periodontal inflammation in older people. However, the impact of oral dysbiosis on periodontal inflammation has not been fully elucidated. Therefore, further studies should focus on clarifying the associations of oral microbiota with PISA.

## Figures and Tables

**Table 1:** Periodontal conditions in older people.

Periodontal Conditions	Number of Patients (%)
Probing depth	
<4 mm	36 (36.0%)
≥4 mm and <6 mm	32 (32.0%)
≥6 mm	32 (32.0%)
≥4 mm periodontal pocket with BOP	
No	62 (62.0%)
Yes	38 (38.0%)
≥6 mm periodontal pocket with BOP	
No	77 (77.0%)
Yes	23 (23.0%)

**Table 2:** Associations of clinical parameters with periodontal inflamed surface area (PISA) and periodontal epithelial surface area (PESA).

Clinical Factor (*n*)	PISA	*p*-Value	PESA	*p*-Value
Age in years				
60–69 (42)	203.8 ± 172.5	0.70 ^a^	1180.8 ± 207.2	0.40 ^a^
70–79 (37)	208.0 ± 313.4		1086.5 ± 361.0	
80–89 (20)	191.4 ± 268.0		1062.0 ± 435.3	
90–99 (1)	165.5		1036.3	
Sex				
Men (44)	208.2 ± 271.2	0.83 ^b^	1122.9 ± 367.4	0.68 ^b^
Women (56)	197.9 ± 231.9		1119.0 ± 323.4	
Cardiovascular disease				
No (91)	208.1 ± 258.0	0.99 ^b^	1126.0 ± 349.0	0.61 ^b^
Yes (9)	145.1 ± 113.7		1067.5 ± 267.0	
Hypertension				
No (75)	191.8 ± 235.7	0.52 ^b^	1100.2 ± 358.9	0.30 ^b^
Yes (25)	234.4 ± 287.1		1182.3 ± 281.1	
Diabetes				
No (87)	185.0 ± 207.3	0.32 ^b^	1115.0 ± 334.5	0.88 ^b^
Yes (13)	319.2 ± 430.8		1158.6 ± 399.1	
Dyslipidemia				
No (79)	207.6 ± 264.1	0.91 ^b^	1110.3 ± 363.8	0.39 ^b^
Yes (21)	183.0 ± 183.3		1159.1 ± 244.4	
Smoking				
Non-smoker (90)	183.7 ± 221.2	0.15 ^b^	1099.2 ± 332.2	0.04 ^b^
Current/former smoker (10)	371.2 ± 401.7		1314.5 ± 382.4	
Number of remaining teeth				
≥20 (81)	221.4 ± 265.2	0.07 ^a^	1210.4 ± 286.7	<0.01 ^a^
10–19 (17)	134.4 ± 141.0		802.7 ± 232.2	
0–9 (2)	12.5 ± 17.7		191.1 ± 139.5	

^a^ Kruskal–Wallis test. ^b^ Mann–Whitney *U* test. *p*-values < 0.05 were considered statistically significant.

**Table 3:** Associations of *P. gingivalis* presence with PISA and PESA in 72 propensity score-matched patients.

Clinical Factor (*n*)	*P. gingivalis*		*p*-Value
	Negative (36)	Positive (36)	
Age	72.1 ± 7.9	72.1 ± 7.7	0.93 ^a^
Age in years			
60–69 (32)	15 (41.7%)	17 (47.2%)	0.77 ^b^
70–79 (27)	15 (41.7%)	12 (33.3%)	
80–89 (13)	6 (16.7%)	7 (19.4%)	
Sex			
Men (30)	13 (36.1%)	17 (47.2%)	0.43 ^c^
Women (42)	23 (63.9%)	19 (52.8%)	
Cardiovascular disease			
No (65)	33 (91.7%)	32 (88.9%)	0.50 ^c^
Yes (7)	3 (8.3%)	4 (11.1%)	
Hypertension			
No (54)	28 (77.8%)	26 (72.2%)	0.39 ^c^
Yes (18)	8 (22.2%)	10 (27.8%)	
Diabetes			
No (66)	32 (88.9%)	34 (94.4%)	0.34 ^c^
Yes (6)	4 (11.1%)	2 (5.6%)	
Dyslipidemia			
No (58)	29 (80.6%)	29 (80.6%)	0.62 ^c^
Yes (14)	7 (19.4%)	7 (19.4%)	
Smoking			
Non-smoker (66)	34 (94.4%)	32 (88.9%)	0.34 ^c^
Current/former smoker (6)	2 (5.6%)	4 (11.1%)	
Remaining teeth	24.0 ± 6.1	23.2 ± 5.1	0.22 ^a^
PISA (mm^2^)	149.5 ± 134.0	199.0 ± 292.0	0.36 ^a^
PESA (mm^2^)	1093.8 ± 345.5	1113.7 ± 334.8	0.81 ^a^

^a^ Mann–Whitney U test. ^b^ χ^2^ test. ^c^ Fisher’s exact test. *p*-values <0.05 were considered statistically significant.

**Table 4:** Associations of *P. gingivalis*/*T. forsythia* presence with PISA and PESA in 72 propensity score-matched patients.

Clinical Factor (*n*)	*P. gingivalis*/*T.forsythia* Positive		*p*-Value
	No (36)	Yes (36)	
Age	72.1 ± 8.3	71.9 ± 7.0	0.91 ^a^
Age in years			
60–69 (31)	16 (44.4%)	15 (41.7%)	0.88 ^b^
70–79 (28)	13 (36.1%)	15 (41.7%)	
80–89 (13)	7 (19.4%)	6 (16.7%)	
**Sex**			
Men (29)	15 (41.7%)	14 (38.9%)	0.50 ^c^
Women (43)	21 (58.3%)	22 (61.1%)	
Cardiovascular disease			
No (68)	33 (91.7%)	35 (97.2%)	0.31 ^c^
Yes (4)	3 (8.3%)	1 (2.8%)	
Hypertension			
No (56)	28 (77.8%)	28 (77.8%)	0.61 ^c^
Yes (16)	8 (22.2%)	8 (22.2%)	
Diabetes			
No (60)	31 (86.1%)	29 (80.6%)	0.38 ^c^
Yes (12)	5 (13.9%)	7 (19.4%)	
Dyslipidemia			
No (58)	29 (80.6%)	29 (80.6%)	0.62 ^c^
Yes (14)	7 (19.4%)	7 (19.4%)	
Smoking			
Non-smoker (66)	34 (94.4%)	32 (88.9%)	0.34 ^c^
Current/former smoker (6)	2 (5.6%)	4 (11.1%)	
Remaining teeth	23.6 ± 6.2	23.0 ± 5.1	0.91 ^a^
PISA (mm^2^)	150.4 ± 136.8	274.1 ± 347.5	0.53 ^a^
PESA (mm^2^)	1073.6 ± 351.5	1144.4 ± 377.5	0.41 ^a^

^a^ Mann–Whitney U test. ^b^ χ^2^ test. ^c^ Fisher’s exact test. *p*-values <0.05 were considered statistically significant.

**Table 5:** Associations of *T. forsythia*/*T. denticola* presence with PISA and PESA in 72 propensity score-matched patients.

Clinical Factor (*n*)	*T. forsythia*/*T. denticola* Positive		*p*-Value
	No (36)	Yes (36)	
Age	71.7 ± 7.5	71.1 ± 7.8	0.71 ^a^
Age in years			
60–69 (35)	17 (47.2%)	18 (50.0%)	0.97 ^b^
70–79 (25)	13 (36.1%)	12 (33.3%)	
80–89 (12)	6 (16.7%)	6 (16.7%)	
Sex			
Men (30)	16 (44.4%)	14 (38.9%)	0.41 ^c^
Women (42)	20 (55.6%)	22 (61.1%)	
Cardiovascular disease			
No (67)	33 (91.7%)	34 (94.4%)	0.50 ^c^
Yes (5)	3 (8.3%)	2 (5.6%)	
Hypertension			
No (56)	28 (77.8%)	28 (77.8%)	0.61 ^c^
Yes (16)	8 (22.2%)	8 (22.2%)	
Diabetes			
No (65)	33 (91.7%)	32 (88.9%)	0.50 ^c^
Yes (7)	3 (8.3%)	4 (11.1%)	
Dyslipidemia			
No (61)	31 (86.1%)	30 (83.3%)	0.50 ^c^
Yes (11)	5 (13.9%)	6 (16.7%)	
Smoking			
Non-smoker (64)	33 (91.7%)	31 (86.1%)	0.36 ^c^
Current/former smoker (8)	3 (8.3%)	5 (13.9%)	
Remaining teeth	23.1 ± 5.8	23.3 ± 5.2	0.95 ^a^
PISA (mm^2^)	130.0 ± 134.2	259.6 ± 286.3	0.02 ^a^
PESA (mm^2^)	1014.1 ± 318.6	1187.2 ± 347.6	0.03 ^a^

^a^ Mann–Whitney *U* test. ^b^ χ^2^ test. ^c^ Fisher’s exact test. *p*-values < 0.05 were considered statistically significant.

## Data Availability

All data generated or analyzed in this study are included in this article.

## Supplementary Materials

The following are available online at https://www.mdpi.com/article/10.3390/diagnostics11081397/s1, Table S1: Multivariable analysis with the presence of *P. gingivalis* as dependent valuable, Table S2: Multivariable analysis with the presence of *T. forsythia* as dependent valuable, Table S3: Multivariable analysis with the presence of *T. denticola s* as dependent valuable, Table S4: Multivariable analysis with the presence of *P. gingivalis*/*T. forsythia* as dependent valuable, Table S5: Multivariable analysis with the presence of *P. gingivalis*/*T. denticola* as dependent valuable, Table S6: Multivariable analysis with the presence of *T. forsythia*/*T. denticola* as dependent valuable, Table S7: Multivariable analysis with the presence of *P. gingivalis*/*T. forsythia*/*T. denticola* as dependent valuable, Table S8: Multivariable analysis with the presence of *C. albicans* as dependent valuable, Table S9: Multivariable analysis with the presence of *P. gingivalis*/*C. albicans* as dependent valuable, Table S10: Multivariable analysis with the presence of *T. forsythia*/*C. albicans* as dependent valuable, Table S11: Multivariable analysis with the presence of *T. denticola*/*C. albicans* as dependent valuable, Table S12: Multivariable analysis with the presence of *P. gingivalis*/*T. forsythia*/*C. albicans* as dependent valuable, Table S13: Multivariable analysis with the presence of *P. gingivalis*/*T. denticola*/*C. albicans* as dependent valuable, Table S14: Multivariable analysis with the presence of *T. forsythia*/*T. denticola*/*C. albicans* as dependent valuable, Table S15: Multivariable analysis with the presence of *P. gingivalis*/*T. forsythia*/*T. denticola*/*C. albicans* as dependent valuable.
