# Supplementary material for: The Associations of Periodontopathic Bacteria and Oral Candida with Periodontal Inflamed Surface Area in Older Adults Receiving Supportive Periodontal Therapy
Source: Diagnostics (Basel). 2021 Aug 2;11(8):1397. doi: 10.3390/diagnostics11081397 (PMC8392537; doi:10.3390/diagnostics11081397)
Supplement: Supplementary file 1 [file diagnostics-11-01397-s001.zip › diagnostics-1300204-supplementary.pdf]

Supplementary Table S1. Multivariable analysis with the presence of *P. gingivalis* as dependent valuable

Variables	Odds ratio	95% CI	P
Remaining teeth	0.95	0.88-1.03	0.20

Supplementary Table S2. Multivariable analysis with the presence of *T. forsythia* as dependent valuable

Variables	Odds ratio	95% CI	P
Age	0.94	0.87-1.00	0.07
PISA	1.00	1.00-1.01	0.10
PESA	1.00	1.00-1.003	0.54

Supplementary Table S3. Multivariable analysis with the presence of *T. denticola s* as dependent valuable

Variables	Odds ratio	95% CI	P
PESA	1.00	1.00-1.003	0.048

Supplementary Table S4. Multivariable analysis with the presence of *P. gingivalis*/*T. forsythia* as dependent valuable

Variables	Odds ratio	95% CI	P
Remaining teeth	0.95	0.88-1.03	0.21

Supplementary Table S5. Multivariable analysis with the presence of *P. gingivalis*/*T. denticola* as dependent valuable

Variables	Odds ratio	95% CI	P
PISA	1.00	1.00-1.003	0.48
PESA	1.00	1.00-1.003	0.24
Dyslipidemia	0.41	0.13-1.38	0.15

Supplementary Table S6. Multivariable analysis with the presence of *T. forsythia*/*T. denticola* as dependent valuable

Variables	Odds ratio	95% CI	P
PISA	0.97	0.92-1.02	0.24
PESA	1.00	0.998-1.00	0.58
Dyslipidemia	1.00	1.00-1.003	0.73

Supplementary Table S7. Multivariable analysis with the presence of *P. gingivalis*/*T. forsythia*/*T. denticola* as dependent valuable

Variables	Odds ratio	95% CI	P
PISA	1.00	1.00-1.004	0.27
PESA	1.00	1.00-1.002	0.42

Supplementary Table S8. Multivariable analysis with the presence of *C. albicans* as dependent valuable

Variables	Odds ratio	95% CI	P
Hypertension	0.39	0.12-1.29	0.12
Dyslipidemia	0.39	0.11-1.43	0.15
Smoking	3.32	0.78-14.2	0.10

Supplementary Table S9. Multivariable analysis with the presence of *P. gingivalis*/*C. albicans* as dependent valuable

Variables	Odds ratio	95% CI	P
Hypertension	0.46	0.12-1.76	0.26
Remaining teeth	0.91	0.83-0.99	0.03

Supplementary Table S10. Multivariable analysis with the presence of *T. forsythia*/*C. albicans* as dependent valuable

Variables	Odds ratio	95% CI	P
Hypertension	0.38	0.10-1.42	0.15
Dyslipidemia	0.34	0.08-1.46	0.15
Smoking	4.41	0.97-20.1	0.06
PISA	1.00	1.00-1.002	0.70

Supplementary Table S11. Multivariable analysis with the presence of *T. denticola*/*C. albicans* as dependent valuable

Variables	Odds ratio	95% CI	P
Diabetes	0.23	0.02-2.20	0.20
Dyslipidemia	0.33	0.07-1.57	0.16
PISA	1.00	1.00-1.003	0.34

Supplementary Table S12. Multivariable analysis with the presence of *P. gingivalis*/*T. forsythia*/*C. albicans* as dependent valuable

Variables	Odds ratio	95% CI	P
Remaining teeth	0.91	0.83-0.99	0.03
PISA	1.00	1.00-1.003	0.19

Supplementary Table S13. Multivariable analysis with the presence of *P. gingivalis*/*T. denticola* /*C. albicans* as dependent valuable

Variables	Odds ratio	95% CI	P
Dyslipidemia	0.27	0.03-2.22	0.22
PISA	1.00	1.00-1.004	0.16

Supplementary Table S14. Multivariable analysis with the presence of *T. forsythia*/*T. denticola*/*C. albicans* as dependent valuable

Variables	Odds ratio	95% CI	P
Dyslipidemia	0.14	0.02-1.12	0.06
PISA	1.00	1.00-1.003	0.36

Supplementary Table S15. Multivariable analysis with the presence of *P. gingivalis*/*T. forsythia*/*T. denticola*/*C. albicans* as dependent valuable

Variables	Odds ratio	95% CI	P
Dyslipidemia	0.27	0.03-2.23	0.22
PISA	1.00	1.00-1.003	0.34
